# The Physiology and Pathology of the Mind

**Published:** 1867-09

**Authors:** 


The Physiology and Pathology of the Mind. By HENRY MAUDSLEY, M.D., London; Physician to the West London Hospital; Honorary Member of the Medico-Psycological Society of Paris; formerly Resident Physician to the Manchester Royal Lunatic Hospital, etc., etc. New York: D. APPLETON & Co., 443 and 445 Broadway. 1867.

This is an elegantly published octavo volume of 442 pages. It is divided into two parts. The first part relates to the "Physiology of Mind," and embraces nine chapters, as follows:

1. On the Method of the Study of Mind.
2. The Mind and the Nervous System.
3. The Spinal Cord, or Tertiary Nervous Centres, as centres of reflex action.
4. Secondary Nervous Centres, or Sensory Ganglia.
5. Hemispherical Ganglia; Certical Cells of the Cerebral Hemisphere, etc.
6. The Emotions.
7. Volition.
8. Motor Nervous Centres, or Motorium Communi.
9. Memory and Imagination.

Part Second treats of the "Pathology of the Mind;" and is divided into seven sections, as follows:

1. On the Causes of Insanity.
2. On the Insanity of Early Life.
3. The Varieties of Insanity.
4. The Pathology of Insanity.
5. The Diagnosis of Insanity.
6. The Prognosis of Insanity.
7. The Treatment of Insanity.

The author of this work has made a systematic attempt to elucidate the pathology and treatment of mental diseases, by the principles and facts of physiology. The general scope of the work is sufficiently indicated by the table of contents, which we have quoted. The author says, in his preface, "The aim which I have had in view throughout this work has been twofold; first, to treat of mental phenomena from a physiological rather than from a metaphysical point of view; and, secondly, to bring the manifold instructive instances presented by the unsound mind to bear upon the interpretation of the obscure problems of mental science."

We think every reader will find it an interesting and instructive book.

For sale by S. C. GRIGGS & Co., 39 and 41 Lake Street. Price, $4.00.

